# Preclinical evidence for supra-clinical acute kidney injury: current biomarkers are not enough

**DOI:** 10.1186/s40635-026-00965-7

**Published:** 2026-08-26

**Authors:** Justine Serre, David Legouis, Sandrine Placier, Charles Verney, Wionna Desvarieux, David Buob, Juliette Hadchouel, Pierre Galichon

**Affiliations:** 1 Inserm, Sorbonne Université, Common and Rare Kidney Diseases: from Molecular Events to Precision Medicine CoRaKiD, Paris, F-75020 France; 2 Service de Néphrologie et Dialyse, Tenon hospital, Assistance Publique – Hôpitaux de Paris, Paris, France; 3 Division of Intensive Care Medicine, Department of Anesthesiology, Clinical Pharmacology, Critical Care and Emergency Medicine, Geneva University Hospital, Geneva, Switzerland; 4 Laboratory of Nephrology, Department of Physiology and Cell Metabolism, University of Geneva, Geneva, Switzerland; 5 Department of Pathology, Pitié Salpêtrière Hospital, Assistance Publique-Hôpitaux de Paris (AP-HP), Paris, France; 6 Service de Néphrologie Hôpital Ambroise Paré, Assistance Publique – Hôpitaux de Paris, Université Versailles — Saint-Quentin-en-Yvelines, Université Paris-Saclay, Boulogne-Billancourt, France

**Keywords:** AKI, Supra-clinical AKI, Biomarkers

## Abstract

**Background:**

Acute kidney injury (AKI) is a frequent complication in hospitalized patients, affecting up to 50% of those in intensive care units (ICU). It is linked to high morbidity and mortality, with severity closely tied to patient outcomes. While early biomarkers like KIM1 and NGAL can detect subclinical AKI, their practical benefit is limited, as treatment must begin at suspicion rather than biomarker confirmation. Conversely, when injury exceeds serum creatinine’s diagnostic capacity, a “blind spot” emerges, highlighting the need for “supra-clinical” AKI biomarkers. The KDIGO staging system, based on urine output and creatinine, lacks granularity: even in stage 3, outcomes range from full recovery to permanent dialysis. Anuric patients, despite complete loss of filtration, also show heterogeneous recovery, suggesting injury severity influences prognosis. This study aims to explore “supra-clinical” AKI and assess whether further injury occurs beyond current clinical markers using a preclinical ischemia-reperfusion model and human single-cell transcriptomic data.

**Results:**

We performed unilateral renal ischemia of increased duration (5-30 minutes), associated with contralateral nephrectomy, in mice. Plasma urea and creatinine concentration reached a plateau when ischemia duration exceeded 10 minutes. In contrast, we observed a continuous increase in the severity of the renal lesions with the ischemia duration. Similarly, the expression level of Quinolinate phosphoribosyltransferase (QPRT), an enzyme contributing to the biosynthesis of nicotinamide adenine dinucleotide (NAD), was inversely correlated to the ischemia duration. Using single-nucleus RNA sequencing data on human kidney biopsies from ICU patients, we showed a largely reciprocal distribution of QPRT and KIM1 (Kidney Injury Marker 1), expressed specifically in injured proximal tubule cells.

**Conclusions:**

Our data demonstrate the existence of a “supra-clinical” stage in AKI, where more ischemia causes more histological and metabolic injury than serum creatinine, serum urea and KIM1 expression can reflect. This highlights a critical diagnostic blind spot: there are currently no available biomarkers to distinguish several stages of AKI when the plasma creatinine concentration does not increase further, or in patients treated with dialysis.

**Supplementary Information:**

The online version contains supplementary material available at https://doi.org/10.1186/s40635-026-00965-7.

## Introduction

Acute kidney injury (AKI) is a common complication among hospitalized patients and affects up to 50% of those admitted to intensive care units [1]. AKI is associated with high morbidity and mortality rates, and its severity correlates with mortality [2]. This observation underscores the need to identify patients with the most severe forms of AKI. Recent studies have largely focused on identifying early, subclinical biomarkers of AKI, such as KIM1 (Kidney Injury Molecule 1) and NGAL (Neutrophil gelatinase-associated lipocalin), markers of kidney aggression that are more sensitive than serum creatinine. However, the early diagnosis of subclinical AKI provides little practical benefit, since the bundle of care for AKI must be instated upon suspicion rather than on biomarker confirmation [3].

At the opposite end of the spectrum, we hypothesized that when injury surpasses the discrimination capacity of serum creatinine, a diagnostic blind spot appears, underscoring the need for biomarkers of “supra-clinical” AKI. Clinical outcomes among patients classified as KDIGO stage 3 range from full recovery to permanent dependence on renal replacement therapy, underscoring the limited granularity of the KDIGO classification [1]. This staging system relies exclusively on changes in urine output, serum creatinine and renal replacement therapy initiation. Yet, even within the most severe category (KDIGO 3), patients fulfilling both criteria have significantly worse outcomes than those meeting only one [4, 5]. Moreover, renal outcomes among anuric patients, despite anuria indicating a complete loss of glomerular filtration, remain highly heterogeneous, ranging from full recovery to irreversible end-stage kidney disease. The severity of the insult might be a determinant of recovery after AKI, as suggested by the independent association of cardiopulmonary resuscitation time with recovery of AKI occurring after circulatory arrest [6].

Such heterogeneity illustrates the need for additional biomarkers capable of identifying ongoing or worsening injury even in patients already meeting KDIGO stage 3 criteria.

Our objective was to investigate the existence of “supra-clinical” AKI and to determine whether incremental injury can occur beyond the dynamic range of current clinical markers, such as when serum creatinine levels reach a plateau. For this purpose, we used a preclinical model of ischemia-reperfusion-induced AKI with graded ischemia duration to assess the dynamic range of clinical and subclinical AKI markers in comparison with histological and metabolic markers. To further characterize the biological relevance of these “supra-clinical” histological changes, we investigated the expression of two molecules involved in energy metabolism, a key pathway in the pathophysiology of acute tubular injury: PGC1α, a regulator of mitochondrial biogenesis, and Quinolinate phosphoribosyltransferase (QPRT), an enzyme contributing to the biosynthesis of nicotinamide adenine dinucleotide (NAD), a key component in major metabolic processes such as oxidative phosphorylation and DNA processing.;Finally, by integrating single-cell transcriptomic data from AKI patients, we placed these findings in a clinical context to assess the residual metabolic activity of injured human kidneys.

## Methods

### Animals studies

#### Animals

We used 8-week-old (24–30 g) C57Bl/6J males from Charles River Laboratories (Saint-Germain-sur-l’Arbresle, France). They were maintained under routine vivarium conditions, in a pathogen-free animal facility, in ventilated cages with a 12/12 photoperiod, at 22 ± 2 °C with a 55 ± 10% humidity. Food and water were provided ad libitum. The study was approved by the ‘Darwin Ethics Committee’ of Sorbonne University and the Ministère de l’Enseignement Supérieur et de la Recherche (authorization APAFIS#27761–2020102120323006 delivered on 19 January 2021).

#### Induction of ischemia with the RIRI device

Unilateral renal ischemia-reperfusion injury was performed using the RIRI device as previously described [7]. The mouse was anaesthetised with an intraperitoneal injection of a mix containing ketamine and xylazine (90 mg/kg and 8 mg/kg, respectively). A right nephrectomy was performed. In the same surgery time, the left renal vascular pedicle was dissected. The thread of the RIRI clamp was placed around the pedicle with the two cylinders on each side of the pedicle. The catheter was inserted into a posterior incision with the flexible end towards the kidney. The extremities of the thread were then inserted within the tube of the catheter and exposed through the other end. The solid portion of the catheter was fixed to the skin of the mouse. 24 h after the clamp-positioning surgery, the mouse was sedated by exposure to 2% isoflurane for 1 min on a heating pad. The clamp was closed by pulling the external extremities of the thread to clamp the vascular pedicle for 5, 10, 15, 20 and 30 min. After that time the tension was released to stop the renal ischemia.

We used 10 mice for each ischemia duration. Sham mice (*n* = 7) underwent the same surgical procedure, without the clamping of the renal vasculature.

#### Tissue collection

Twenty-four hours after ischemia, mice were anaesthetized with an intraperitoneal injection of a mix containing ketamine and xylazine (100 mg/kg and 10 mg/kg, respectively). From the retroorbital sinus, 500 µL of blood was collected. The kidneys were then collected. The median part of each kidney was fixed in Formalin–Acetic acid–Alcohol (FAA) overnight and embedded in paraffin for histological examination. The remaining parts were frozen in liquid nitrogen for RNA and protein analysis.

Plasma creatinine and urea concentration was measured with a Konelab enzymatic spectrophotometric analyzer (ThermoFischer Scientific, Waltham, MA, US).

#### Histological analysis

For histological evaluation, 3-micrometre sections were stained with periodic acid–Schiff. The scoring was performed in a masked manner on coded slides by two different investigators, using the following scale: 0: no tubular damage; 1: damage in 1–20% of the corticomedullary tubules; 2: damage in 20–100% of the corticomedullary tubules; 3: same as 2 plus cortical dilatation; 4: same as in 2 plus cortical oedema and zones of infarction.

#### RNA extraction and RT-qPCR

The total RNA was extracted from the mouse kidneys using Tri-reagent and BAN (Molecular Research Center Inc.—Euromedex, Souffelweyersheim, France). The RNA quality was checked by control of optical density at 260 and 280 nm. The contaminating genomic DNA was removed by RNase-free DNAse (Thermo Fisher Scientific, Illkirch, France) for 30 min at 37 °C. The cDNA was synthesized from 1 µg of purified RNA using Maxima Enzyme Mix (Thermo Fisher Scientific, Illkirch, France) according to the manufacturer’s instructions. Real-time PCR amplification was performed using the the light cycler 480 SYBR Green I Master (Roche Life Science) and the Bio-Rad Connect Real-Time detection system apparatus. Analysis was performed using the CFX Maestro 2.2 software (Version 5.2.008.0222). All of the samples were assayed in duplicate, and the average value of the duplicate was used for quantification. The analysis of the relative gene expression (RGE) was performed using the Pfaffl equation [8].The geometric mean of the E^∆Ct^ of 2–3 housekeeping genes (*Gusb*, *Ubc* and *RPL32*) was used as the housekeeping reference. The sequences of primers used in our studies are listed in Table 1.

#### Western blot

Protein lysates from mouse kidneys were prepared with RIPA extraction buffer containing phosphatase and protease inhibitors (Roche). Total protein concentration was measured using the BCA protein assay kit (23225/23227, ThermoFisher Scientific). 20 µg of proteins were loaded onto polyacrylamide electrophoresis gels for separation and transferred onto nitrocellulose membranes. The membranes were blocked with milk and probed with an antibody directed against QPRT (Quinolinate phosphoribosyltransferase − 25174-1-AP, Proteintech). Membranes were then probed with a horseradish peroxidase-conjugated secondary antibody (NA9340, donkey anti-rabbit, Amersham). Bands were visualized by enhanced chemiluminescence (Clarity Western ECL substrate; Bio-Rad, 170–5061). The quantification of total proteins, visualized with the Stain-Free^®^ reagent from Bio-Rad, was used to normalize the loading. A ChemiDoc MP imaging system (12003154, Biorad) was used to reveal bands, and densitometric analysis was used for quantification.

#### Statistical analysis

The statistical test used for the analysis is indicated in the figure legends. A difference between groups was considered significant when *p* < 0.05. The statistical analysis was performed using the GraphPad PRISM software v8 (Ritme, Paris, France). Rolling correlations were calculated using the KendallTauB function of the DescTools package in R (Signorell A (2025). *DescTools: Tools for Descriptive Statistics*. R package version 0.99.60.24, https://github.com/andrisignorell/desctools) and the rollapply function of the zoo packages in R [9].

### Human studies

#### Experimental kidney biopsies

Human snRNA-seq data were obtained from kidney biopsies performed in 5 critically ill patients, as previously described (Legouis et al., iScience, 2024). All patients were admitted to the ICU and biopsies were performed immediately prior to planned withdrawal of resuscitation measures. AKI stage ranged from KDIGO stage 1 to 3 (based on serum creatinine or urine output criteria), and biopsies were obtained 6 to 25 days after AKI diagnosis. The cause of AKI was multifactorial in all cases, combining ischemic and inflammatory (cytokine storm). All 5 patients displayed severe tubular injury at histology, of variable severity, and one patient required renal replacement therapy. After informed consent was obtained, kidney biopsies were performed using 18G automated biopsy guns (Max-Core, BardCare) under real-time ultrasound guidance. Biopsy cores were snap-frozen and stored at − 80 °C until processing. The study was approved by the local ethical committee for human studies of Geneva, Switzerland (CCER 2020 − 00644, Commission Cantonale d’Ethique de la Recherche) and performed according to the Declaration of Helsinki principles.

#### snRNA-seq library preparation and sequencing

Single-nucleus isolation from frozen kidney tissue was performed using a Dounce homogenization–based protocol, as previously described [10]. Single-nucleus transcriptomes were generated using the 10x Genomics Chromium platform. Barcoded gel bead–in–emulsions were produced and libraries were prepared using the Chromium Single Cell 3′ Library & Gel Bead Kit v2 according to the manufacturer’s instructions.

#### Primary snRNA-seq processing and clustering

FASTQ files were processed using Cell Ranger (v3.1.0). Reads were aligned to a pre-mRNA human reference genome (GRCh38, 2020) to generate UMI count matrices incorporating both exonic and intronic reads for downstream analyses.

Single-nucleus RNA-sequencing control data were retrieved from the Gene Expression Omnibus (GEO; accession GSE131882), which includes three early diabetic kidney samples and three control kidney samples profiled by snRNA-seq. Control samples correspond to non-tumor kidney tissue obtained from patients undergoing nephrectomy for a renal mass; only control samples were retained and used for downstream integration.

Downstream analyses were performed in R using Seurat (v4), including normalization, scaling, dimensionality reduction, and clustering. Briefly, each sample was processed independently and nuclei were excluded if they had fewer than 150 detected genes (*nFeature_RNA*), more than four times the absolute median *nFeature_RNA*, or if more than 1% of UMIs mapped to mitochondrial or ribosomal genes. Ambient RNA contamination in human samples was corrected using SoupX, and doublets were identified and removed using DoubletFinder. Seurat standard integration and clustering workflows were applied to mitigate batch effects, and clusters were annotated by manual inspection of canonical lineage marker expression.

#### External datasets and PT-focused secondary analyses

Public human kidney single-cell RNA-seq data from the Kidney Precision Medicine Project (KPMP) were obtained by downloading a pre-processed Seurat object in.*h5Seurat* format from the KPMP atlas. Associated metadata were imported and matched to the Seurat object using experiment identifiers.

#### Downstream analyses

Analyses were restricted to proximal tubule cells, which were subset from each dataset based on the original cell-type annotations. Dimensionality reduction was performed using uniform manifold approximation and projection (UMAP) on the first 30 principal components. For spatial visualization of gene expression at single-cell resolution, Nebulosa was used to compute kernel density estimates of KIM1 and QPRT expression over the UMAP embeddings. Density maps were visualized separately for each gene and in overlaid representations combining both signals.

## Results

### A preclinical model provides evidence of supraclinical kidney injury

**Fig. 1:**
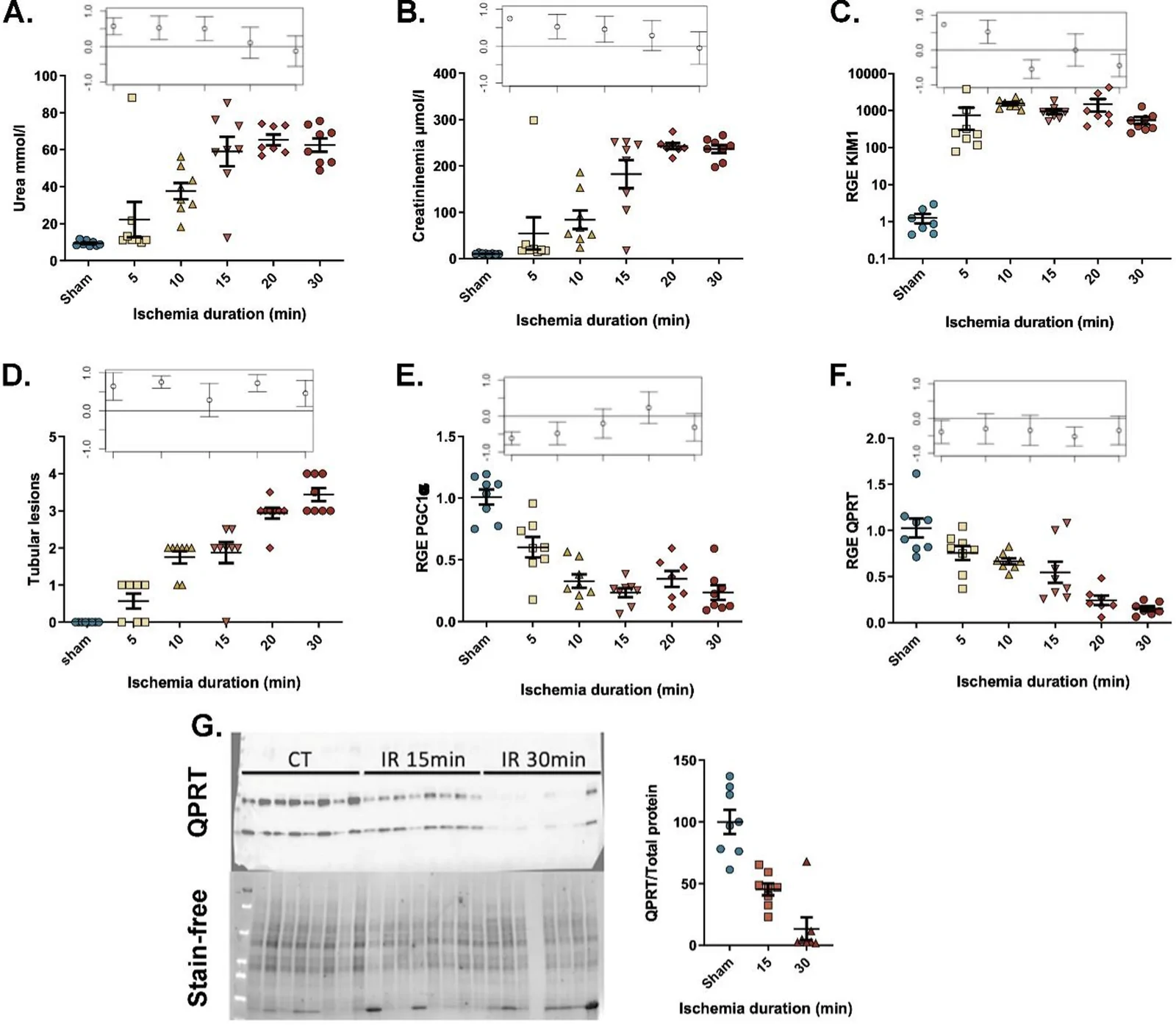
QPRT expression is negatively correlated to the severity of renal ischemia, whereas plasma urea and creatinine reach a plateau when ischemia duration exceeded 10 min. **A-B**. Plasma urea (**A**) and creatinine (**B**) concentration after ischemia of different durations. **C.** Quantification of KIM1 mRNA expression by RT-qPCR in the ischemic mouse kidney. **D.** Histological score following PAS (Periodic acid–Schiff) staining. **E-F.** Quantification of PGC1α and QPRT mRNA expression by RT-qPCR in the ischemic mouse kidney. For panels A-F, Kendall correlations between adjacent groups of ischemia duration are represented by Kendall’s τ with its 95% confidence intervals on the top of each graph. **G.** QPRT protein expression analyzed by western blot in the ischemic mouse kidney. Left panel: representative images of the immunoblot obtained after incubation with an anitbody directed against QPRT (upper panel) or visualization of all proteins in the extract with the Stain-Free reagent (bottom). Right panel: Quantification of QPRT protein expression normalized by total protein extract

We performed a unilateral renal ischemia of increasing duration from 5 to 30 min, combined with contralateral nephrectomy in C57Bl/6J mice. As previously described in this model [7], increases of ischemia over 15 min do not translate into higher serum creatinine and urea concentrations (Fig. 1A-B). The mRNA expression of the subclinical injury marker KIM1 was markedly elevated in the kidney after 5 min of ischemia and further increased after 10 min but failed to reflect further increases in ischemia. Compared to 10 min of ischemia, it even slightly decreased after 15, 20–30 min of ischemia, suggesting a sideration or death of proximal tubular cells (Fig. 1C). Contrasting with these clinical and subclinical markers, the histological severity score increased progressively with the duration of the ischemia (Fig. 1D and Figure S1). Whether these incremental histological lesions relate to structural damage only or to further loss of metabolic activity and viability is unknown.

The expression of PGC1α decreased proportionally to the duration of ischemia of up to 15 min with no further decrease with longer ischemia (Fig. 1E). On the opposite, QPRT mRNA expression decreased linearly with the duration of ischemia, including in the “supra-clinical” phase beyond the dynamic range of urea, creatinine, KIM1 and PGC1α mirroring the incremental increase of the histological score (Fig. 1F). This downregulation was confirmed at the protein level (Fig. 1G).

### KIM1 and QPRT exhibit mutually exclusive expression patterns in proximal tubule cells during AKI

**Fig. 2:**
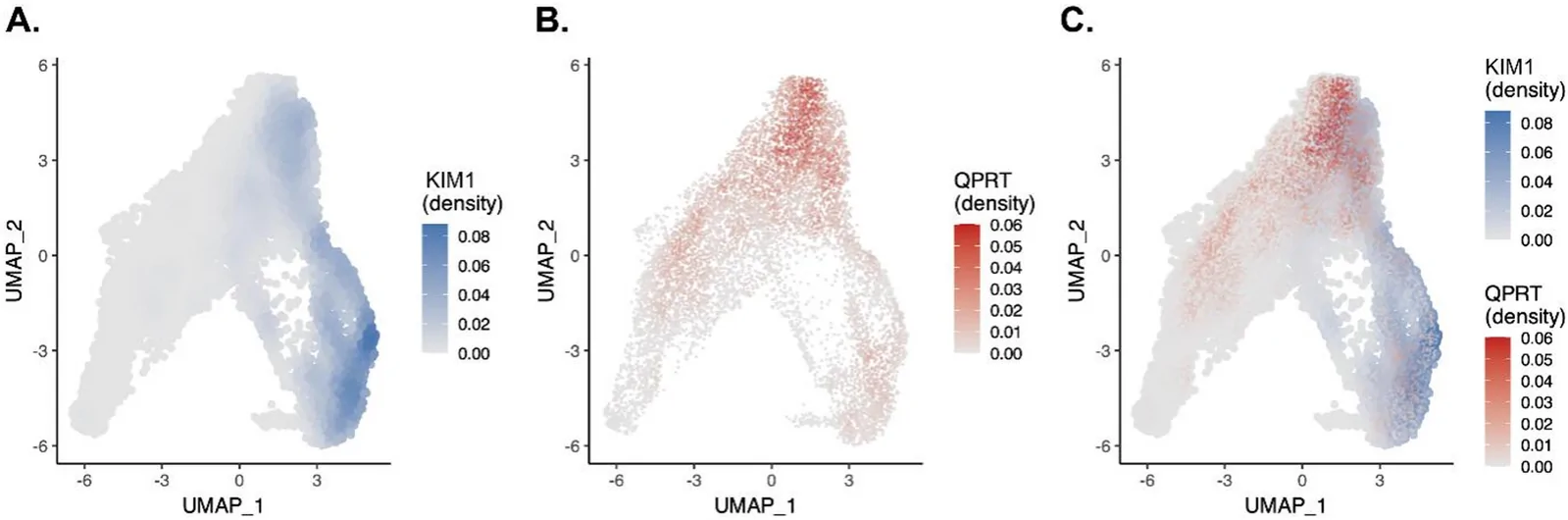
Nebulosa gene-weighted density maps of KIM1 and QPRT expression in proximal tubule cells. UMAP embedding of the ICU AKI dataset (grey background, all cells). Gene expression was visualized using Nebulosa gene-weighted kernel density estimation computed on normalized expression values. **(A)** KIM1 density map. **(B)** QPRT density map. **(C)** Overlaid density representation combining KIM1 (blue) and QPRT (red) on the same UMAP coordinates. Higher color intensity indicates higher estimated local expression density

To assess the relationship between KIM1 and QPRT expression in a human AKI setting, we leveraged kidney biopsies obtained from ICU patients with AKI. We performed snRNA-seq on five renal biopsies and integrated these data with three control samples. Analyses were restricted to the proximal tubule compartment, as KIM-1 expression is confined to PT cells. Consistent with this, KIM1 was detected in a subset of PT cells rather than uniformly across the compartment, consistent with heterogeneous injury-associated cell states. Notably, Nebulosa density mapping on the UMAP revealed a largely reciprocal distribution of KIM1 and QPRT at the single-cell level, with minimal spatial overlap, supporting the existence of distinct PT cell states rather than widespread co-expression. (Fig. 2). This pattern was similarly observed in an independent, publicly available cohort of human PT cells from the KPMP atlas (Figure S2).

## Discussion

We provide evidence that some level of metabolic activity can remain after an ischemic injury causing a complete loss of renal function, and position QPRT as a potential molecular marker of “supra-clinical” AKI severity. Our preclinical data establish QPRT as a marker of injury severity at a single time point but do not address its temporal dynamics during AKI evolution. Such a marker is particularly valuable for patients developing AKI during ICU hospitalization, where the timing of injury is known, rather than patients admitted with established AKI of uncertain duration. In clinical practice, patients may present at various stages of injury progression or resolution. We hypothesize that QPRT expression, or its urinary/circulating surrogates, could inform on residual tubular metabolic capacity regardless of the time elapsed since the initial insult. However, longitudinal studies correlating QPRT expression with clinical trajectory would be required to validate this hypothesis.

QPRT is described as a marker of epithelial metabolic health. However, neither kidney histology nor kidney transcriptomics are likely to be implemented for AKI management due to their invasive nature, highlighting the need for non-invasive markers of supraclinical AKI. Urinary quinolinate/tryptophan (uQ/T) is a new biomarker considered to reflect the acute downregulation of QPRT expression during AKI [11], and could thus be an attractive non-invasive surrogate to kidney QPRT expression, but would not be applicable to anuric patients. It would be interesting to investigate the uQ/T ratio in the blood of AKI patients to determine whether, as in urine, it serves as a reliable marker of QPRT activity. If so, this ratio could also be used in anuric patients. Circulating cell-free DNA is a marker of recent cell death and the involved organ or even cell-type can be determined by the study of their methylation profile [12]. Should this technique be validated in AKI, it could be an excellent surrogate for the histological scoring of acute tubular necrosis.

Our data demonstrate the existence of a “supra-clinical” stage in AKI, where more ischemiacauses more histological and metabolic injury than serum creatinine, serum urea, and KIM1 expression can reflect. This highlights a critical diagnostic blind spot: there are currently no available biomarkers to distinguish several stages of AKI severity when the plasma creatinine concentration does not increase further, or in patients treated with dialysis. Whether such markers could also track ongoing versus receding injury over time remains to be determined in longitudinal studies. Our findings demonstrate that some but not all metabolic markers (i.e., QPRT but not PGC1α expression) can reflect severity stages beyond what currently available markers of function or stress can capture. QPRT might also reach a plateau with longer ischemia durations in mice and could thus become overwhelmed; however, it remains more discriminative than currently available biomarkers. These results advocate for the development of new non-invasive biomarkers capable of detecting the supra-clinical stage of AKI. Based on the human single cell transcriptomic data from AKI patients, we anticipate that such markers will apprehend quantitatively the amount (from 0 to 100%) of injured cells and the extent of their metabolic dysfunction (from normal metabolism to cell death), and thus inform on a large range of severities ranging from normal kidney to irreversible kidney failure.

**Table 1:** Sequence of the primers used for RT-qPCR in mouse kidneys

Transcript	Forward primer	Reverse primer
GusB	CTCTGGTGGCCTTACCTGAT	GAGTTGTTGTCACCTTCACCT
KIM-1	TCAGATTCAAGTCTTGATTTCAGG	CCGCCTTTACTTCCACATAAGAA
PGC1α	GAAAGGGCCAAACAGAGAGA	GTAAATCACACGGCGCTCTT
QPRT	CCGGGCCTCAATTTTGCATC	GGTGTTAAGAGCCACCCGTT
RPL32	GCTGCCATCTGTTTTACGG	TGACTGGTGCCTGATGAA
UBQ	AGCCCAGTGTTACCACCAAG	AGCCCAGTGTTACCACCAAG

## Supplementary Information

Supplementary Material 1

## Data Availability

Data will be made available on reasonable request.

## Abbreviations

AKI: Acute kidney injury
ATN: Acute tubular necrosis
CKD: Chronic kidney disease
ICU: Intensive care unit
KIM1: Kidney Injury Molecule 1
NGAL: Neutrophil gelatinase-associated lipocalin
PAS: Periodic acid Schiff
QPRT: Quinolinate phosphoribosyltransferase
sn-RNAseq: Single-nucleus RNA-sequencing
UMAP: Uniform Manifold Approximation and Projection
