# Correction: Preclinical evidence for supra-clinical acute kidney injury: current biomarkers are not enough

**DOI:** 10.1186/s40635-026-00978-2

**Published:** 2026-09-15

**Authors:** Justine Serre, David Legouis, Sandrine Placier, Charles Verney, Wionna Desvarieux, David Buob, Juliette Hadchouel, Pierre Galichon

**Affiliations:** 1 Inserm, Sorbonne Université, Common and Rare Kidney Diseases: from Molecular Events to Precision Medicine CoRaKiD, Paris, F-75020 France; 2 Service de Néphrologie et Dialyse, Tenon hospital, Assistance Publique – Hôpitaux de Paris, Paris, France; 3 Division of Intensive Care Medicine, Department of Anesthesiology, Clinical Pharmacology, Critical Care and Emergency Medicine, Geneva University Hospital, Geneva, Switzerland; 4 Laboratory of Nephrology, Department of Physiology and Cell Metabolism, University of Geneva, Geneva, Switzerland; 5 Department of Pathology, Pitié Salpêtrière Hospital, Assistance Publique- Hôpitaux de Paris (AP-HP), Paris, France; 6 Service de Néphrologie Hôpital Ambroise Paré, Assistance Publique – Hôpitaux de Paris, Université Versailles — Saint-Quentin-en-Yvelines, Université Paris-Saclay, Boulogne-Billancourt, France

**Correction: Intensive Care Medicine Experimental (2026) 14:113**

**https://doi.org/10.1186/s40635-026-00965-7**

Following publication of the original article [1], the order of the last two authors in the author group has been corrected.

The original article [1] has been updated.
