# Supplementary material for: Preclinical evidence for supra-clinical acute kidney injury: current biomarkers are not enough
Source: Intensive Care Med Exp. 2026 Aug 26;14:113. doi: 10.1186/s40635-026-00965-7 (PMC13518772; doi:10.1186/s40635-026-00965-7)
Supplement: Supplementary file 1 [file 40635_2026_965_MOESM1_ESM.pdf]

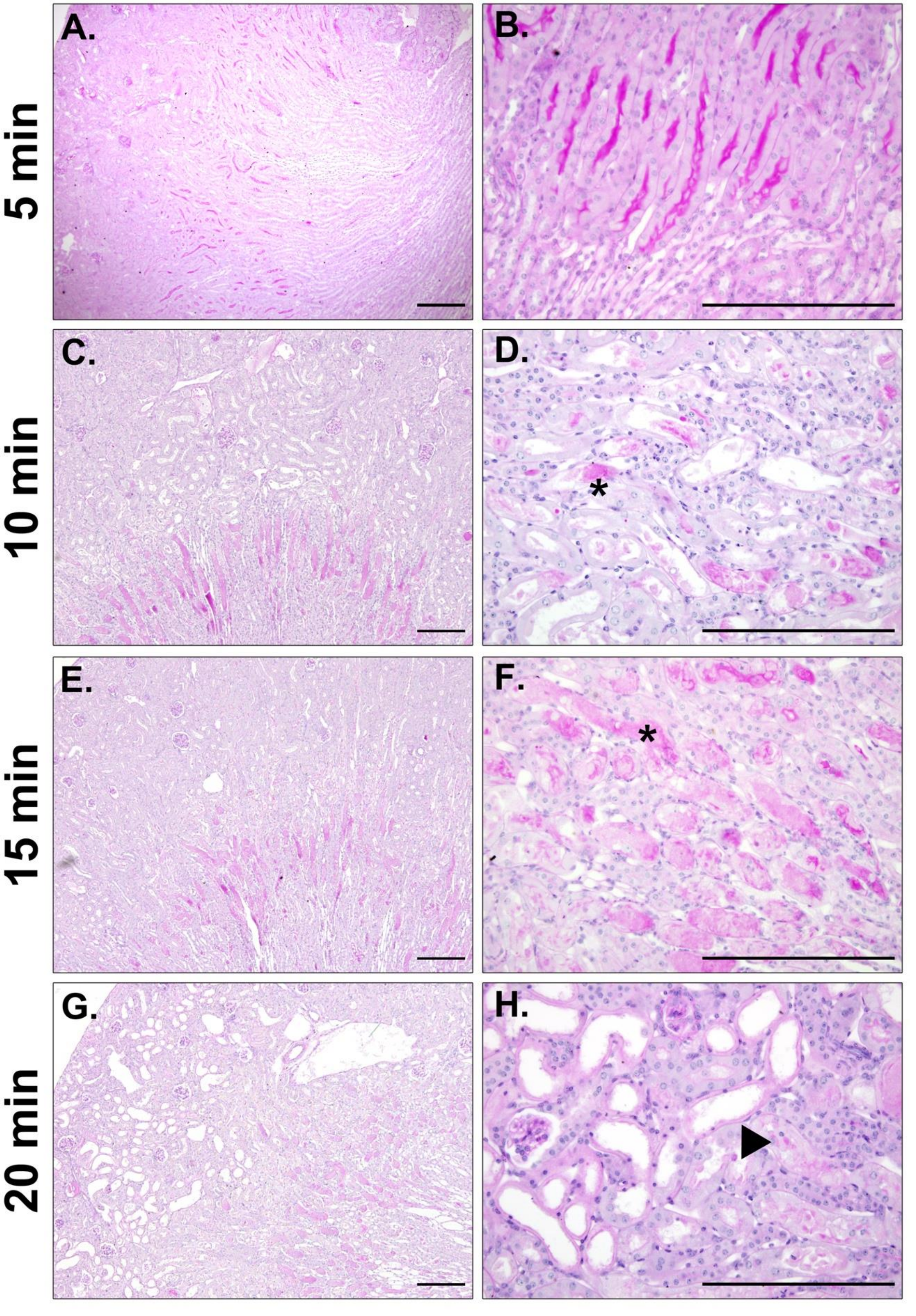

Figure S1. Representative images of the tubular modifications obtained after 5 to 20 min of ischemia. The kidneys were collected 24 h after ischemia and stained with Schiff's periodic acid staining. After a 5-min of ischemia (**A**,**B**), no alteration of renal histology can be observed: the brush border was still visible in all the proximal tubules and no apoptotic cells or casts were observed. After a 10-min (**C**,**D**) or 15-min ischemia (**E**,**F**), a loss of the brush border, a thinning of the epithelium and casts (asterisk) were seen at the corticomedullary junction. After 20 min of ischemia (**G**,**H**), additional cortical dilations could be observed (arrowhead). Scale bar: 200 µm.

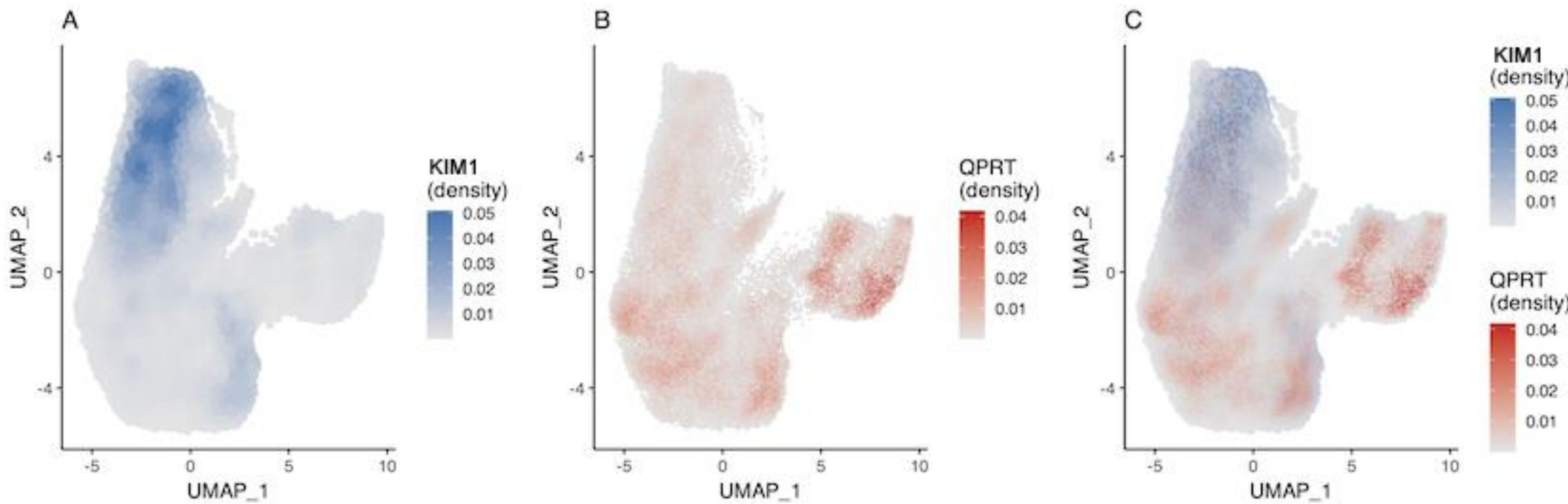

Figure S2: Nebulosa gene-weighted density maps of KIM1 and QPRT expression in proximal tubule cells. UMAP embedding of the KPMP dataset (grey background, all cells). Gene expression was visualized using Nebulosa gene-weighted kernel density estimation computed on normalized expression values. **(A)** KIM1 density map. **(B)** QPRT density map. **(C)** Overlaid density representation combining KIM1 (blue) and QPRT (red) on the same UMAP coordinates. Higher color intensity indicates higher estimated local expression density.
